# Concordance between self- and clinician ratings of depression during inpatient treatment in adolescents: changes over time and probable response shift

**DOI:** 10.1186/s13034-025-00993-3

**Published:** 2025-11-15

**Authors:** Ferdinand Keller, Martin Holtmann, Michael Kölch, Tanja Legenbauer

**Affiliations:** 1 Department of Child and Adolescent Psychiatry, Psychosomatics and Psychotherapy, University Hospital Ulm, Ulm, Germany; 2 LWL University Hospital Hamm for Child and Adolescent Psychiatry, Ruhr-University Bochum, Hamm, Germany; 3 Department of Child and Adolescent Psychiatry and Neurology, Rostock University Medical Center, Rostock, Germany

**Keywords:** Adolescent depression, Self-report, Clinician rating, Concordance, Longitudinal measurement invariance, Response shift

## Abstract

**Background:**

Correlations between self-reported and clinician-rated severity of depression are often only moderate, but studies in adults have shown that they increase over time. This study explored whether a similar effect occurs in adolescents and whether initial agreement vs. disagreement between self- and clinician ratings predicts differential outcomes.

**Method:**

The analyzed data stem from a randomized controlled trial (DeLight) that explored the effect of bright light therapy as an add-on to treatment as usual in an inpatient sample of adolescents (*n* = 224). Depression was assessed at four time points (baseline, 4 weeks, 16 weeks, 28 weeks) using the Beck Depression Inventory (BDI-II) as a self-report measure and the Children’s Depression Rating Scale - Revised (CDRS-R) as a blinded clinician rating.

**Results:**

The correlation between self- and clinician ratings was only moderate at baseline (*r* = .40) but increased considerably to a strong correlation at four weeks (*r* = .72), which was maintained thereafter. The predictive value of initial rater agreement / disagreement for the outcome was small (BDI-II) or non-significant (CDRS-R). Further analyses revealed that the reliability of both instruments increased over time and the factor structure became simpler, with fewer factors and higher factor loadings.

**Conclusions:**

These findings indicate increasing homogeneity within self- and clinician ratings over time and suggest that some type of response shift occurred, with adolescents appearing to increasingly view their depression as a unified concept. A consideration of response shifts could lead to more accurate assessments of treatment effectiveness.

**Supplementary Information:**

The online version contains supplementary material available at 10.1186/s13034-025-00993-3.

## Introduction

When planning clinical studies to evaluate treatment effects, researchers must choose either a self-report or a clinician-based assessment tool as the primary outcome. This choice can be highly relevant, given that trials on depressive disorders have repeatedly demonstrated only moderate correlations between the sum scores based on self- and clinician report [1, 2]. For instance, Sayer et al. [3] found a median correlation of 0.58 between the Beck Depression Inventory (BDI; a self-report instrument) and the Hamilton Depression Rating Scale (HAMD; an observer rating scale) across 12 studies. A more recent meta-analysis by Bukumiric et al. [1] found a pooled correlation of 0.561 between the BDI and HAMD at baseline in studies with adult participants. Hershenberg et al. [4] reported similar values, suggesting a moderate to strong relationship between the two instruments. The Children’s Depression Rating Scale - Revised (CDRS-R), a semi-structured clinical interview, is often used to assess depression severity in clinical trials with children and adolescents. In younger populations, correlations between self- and clinician-rated depression tend to be slightly higher, ranging from 0.46 to 0.80, with Straub et al. [5] reporting a correlation of 0.67 between the BDI-II and the CDRS-R in a predominantly outpatient adolescent sample.

Various potential explanations for the only modest correlation between the HAMD and the BDI have been proposed, including response sets in patients or clinicians, or a different weighting of symptoms between the two instruments, e.g. a supposed stronger emphasis on somatic items in the HAMD and cognitive items in the BDI [1, 3]. Findings on the CDRS-R suggest similar arguments [5]. However, the time point of the assessment appears to have a substantial impact on the strength of the correlation. Sayer et al. [3] found an increase from 0.48 at baseline to 0.77 at the second assessment, and concluded that observer/self-report correlations often increase considerably at later assessment points. The meta-analysis by Bukumiric et al. [1] confirmed and extended these findings: The pooled correlation coefficients between the HAMD and the BDI increased from the aforementioned 0.561 at baseline to 0.774 at the final assessment. Regarding other assessment instruments (mostly the Inventory of Depressive Symptomatology; IDS), rater concordance has also been found to increase over the course of treatment [6–8], although one study reported no increase in (an initially already high) agreement [9]. Dunlop et al. [10, p 177] concluded that “patient-rated measures of depression severity do not correspond strongly with clinician ratings, and are particularly poor prior to the initiation of treatment”. To the best of our knowledge, analogous studies are yet to be conducted in the area of child and adolescent psychiatry.

### Increase in variability of scores and reliability

Several studies have suggested potential methodological reasons for the increase in correlations between depression scales over time (see [1], for an overview and summary), including issues regarding variability of scores and change in reliability over time. More recently, research has also addressed changes in factor structure and has integrated structural equation modeling (SEM) approaches, especially tests of measurement invariance of the instruments over time [10, 11].

**Fig. 1:**
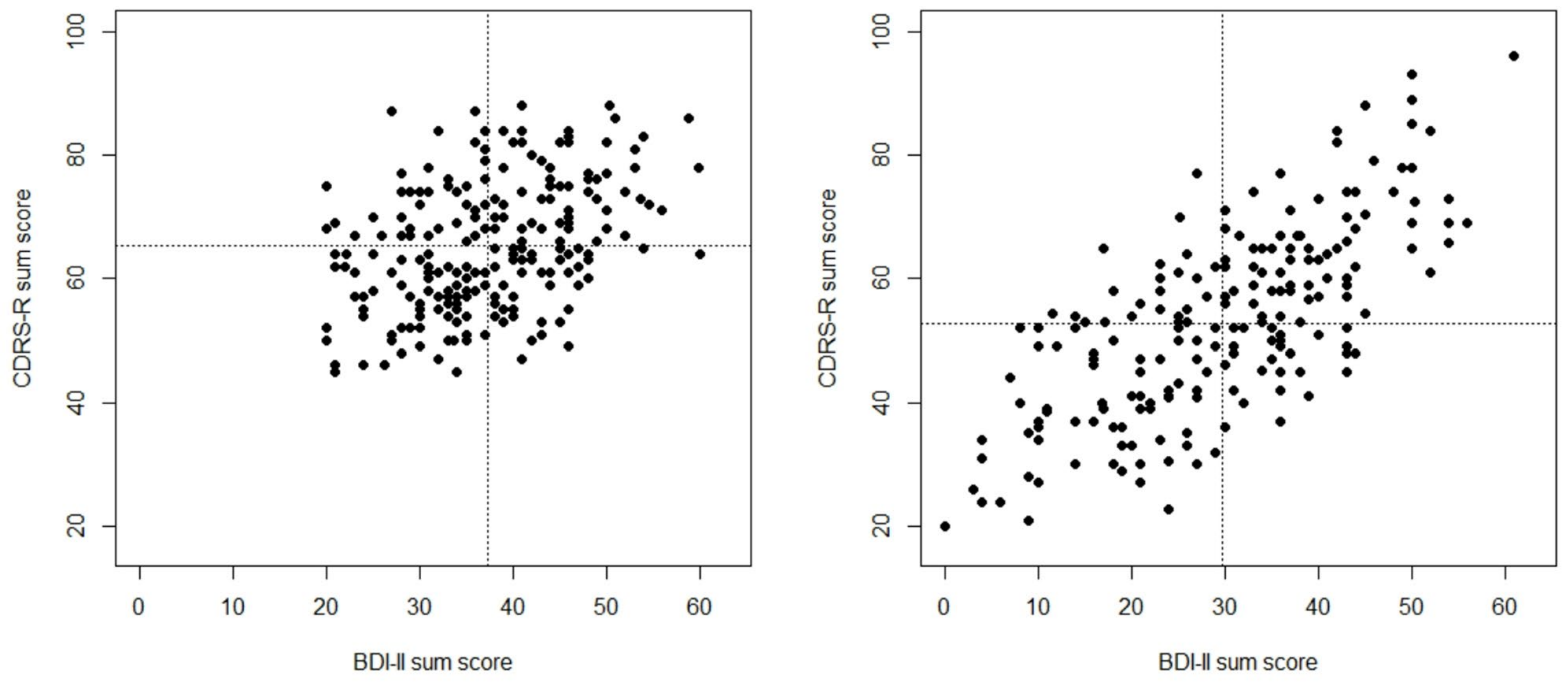
Scatter plot for CDRS-R and BDI-II sum scores at baseline (left side) and after four weeks (right side); the dotted lines indicate mean values

Since most correlation analyses stem from treatment studies, they are commonly characterized by a restricted range of depression scores due to the cut-off criteria imposed for study inclusion. Thus, the variability of depression scores at baseline is necessarily reduced (see Fig. 1 for an illustration with the present data). At later assessment points, the variability of scores is increased due to differential treatment responses, and the scatter plot usually becomes much more widely distributed (see Fig. 1); graphically, the rounder and more condensed scatter plot becomes more elliptic, implying a larger correlation. An increase in variability of depression scores has been found to predict increased correlations [1].

A related phenomenon is increasing homogeneity of the scales over time. Clinically improved patients broaden the range of scores, resulting in higher internal consistency, because discrimination among patients is likely to be more reliable when the range is from asymptomatic to severely depressed than from moderately to severely depressed [3]. Bukumiric et al. [1] identified only five studies that reported repeated reliability coefficients, and were thus unable to analyze reliability coefficients as a predictor in their meta-analysis; however, four of the five studies reported an increase over time. A further study using the BDI revealed an increase from alpha = 0.78 at baseline to 0.92 after 16 weeks of treatment [10]. By contrast, Elhai et al. [12] reported comparable alphas of 0.92 at baseline and 0.93 at follow-up (one month after baseline) in a large sample of adult inpatients. Studies with other instruments likewise revealed increasing reliabilities across repeated assessments (e.g., Stochl et al., [13], for the PHQ-9). This increase in reliability coefficients is not merely a psychometric byproduct, but also suggests that patients may monitor their symptoms more consistently and “homogeneously” over the course of treatment.

Concerning adolescents and focusing on the BDI-II, slightly increasing internal consistencies were found in adolescent inpatients [14] and in a Taiwanese sample of early adolescents suffering from depression [15], but not in an adolescent sample (junior high school students) assessed using the BDI-II in six waves over three years [16]. Regarding the CDRS-R, Mayes et al. [17] reported a clear increase in Cronbach’s alpha from 0.74 at baseline to 0.92 at treatment exit in a clinical sample of adolescents. A similar difference was observed in a depression treatment study [18], in which the alpha increased from 0.65 at baseline to 0.91 at treatment exit (12 weeks); interestingly, the alpha had already increased to 0.81 after one week, and lay at 0.89 after four weeks.

### Factor structure and change over time

The BDI-II is usually seen as unidimensional or as consisting of two or three factors [19, 20]. These factors are highly correlated, and the superior fit of a bifactor model also supports the assumption of a strong general factor in the BDI-II in adolescents [19]. Knowledge about the factor structure of the CDRS-R is much more limited. Guo et al. [21] suggested five factors, although some of these seem questionable, as they mostly consist of only two or three items, and only one factor comprised four items. In an analysis of study exit data in a sample of children, Jain et al. [22] found three factors, but they provided no factor loadings or composition. Isa et al. [18] performed factor analyses over the course of treatment sessions in adolescents. Consistent with the initially lower alphas, five or three factors were required for baseline and for the first two therapy sessions, while a two-factor solution was sufficient thereafter [18]. In a systematic review of the measurement properties of the CDRS-R, Stallwood et al. [23] concluded that the instrument is not unidimensional. However, the authors only found a small number of studies, which yielded factors that differed in number and content, and confirmatory factor analyses are lacking. In German samples (not included in [23]), we found a solution with two factors, comprising the verbal and the non-verbal items, that appeared convincing in both a clinical and a school sample [24, 25]. In summary, factor analysis of CDRS-R data has consistently generated a complex and difficult-to-interpret structure of at least three factors at baseline assessment. However, when administered over additional sessions or at study end, the CDRS-R structure tends to simplify to two factors [18, 23], which reflect (1) reported symptoms of depression and (2) “non-verbal”, clinician-observed signs. The potentially changing factor structure reported by Isa et al. [18], however, questions the (implicit) assumption that the measurement of the construct is entirely comparable over time (longitudinal measurement invariance).

### Correlations on the subscale and item level

Earlier work on the correlation between self- and clinician-rated depression scores at the subscale level did not reveal the expected superiority over the correlations between the total scores [3, 5] (see supplement 1 for details). Analyses on the item level are rare, although a closer look at the correlations between the CDRS-R and BDI-II on the item level seem to encourage their use in clinical practice, and items addressing the same symptom of depression showed moderate to strong correlations [5]. Furthermore, within-person changes in specific items of the CDRS-R were found to predict subsequent depressive episodes [26].

### Predictive value of concordance vs. discordance for improvement

From a clinical perspective (and beyond solely methodological considerations), it seems worthwhile to explore whether initial agreement vs. disagreement between self- and clinician-based assessment leads to differences in outcomes, and thus whether subgroups of concordance/discordance are predictive of treatment outcome. Moreover, factors that contribute to discordance have been evaluated.

Two strategies have been used to define subgroups of concordance/discordance. Sayer et al. [3] suggested building subgroups with scores below vs. above the median of each of the two assessment instruments (HAMD and BDI) at baseline, thus yielding four subgroups. This approach has not been followed in further studies; its rationale and the results for the current study are described in the supplement 1. Dorz et al. [6] proposed distinguishing between individuals who over-rate vs. under-rate their symptoms relative to clinician ratings, an approach which was adopted in other studies [4, 5, 8]. To define the subgroups, self- and clinician-rated scores were both standardized to z-scores; based on the difference between these two scores, patients were classified into under-, equal-, and over-reporters (see Method section below for details). Applying this classification to patient data from a large psychopharmacological trial with adults (PREVENT), Dunlop et al. [8] found some statistically significant relations over the different phases of treatment, e.g., over-raters showed a slower onset of response to acute treatment, but no differences emerged for remission or response. The authors concluded that “poor agreement between patient- and clinician-ratings of depression severity is primarily a state phenomenon, although it is trait-like for some patients” [8, p 96].

### Main research questions

The current study used data from a clinical trial that explored the effect of bright light therapy as an add-on to treatment as usual in an adolescent inpatient sample [27, 28]. The main assessment instruments were the BDI-II (primary outcome) and the (blinded) CDRS-R. We explored the following research questions:

1. What is the correlation between self- and clinician-rated depression at baseline, and is it comparable to the correlations reported in previous studies with adults and adolescents?
2. Does the correlation between self- and clinician rating increase over the assessment time points, as reported in studies with adults?
3. Does initial concordance/discordance predict therapy outcome (BDI-II and CDRS-R)?

As a fourth, exploratory research question, we further investigated potential methodological reasons for the correlational findings over time by analyzing the CDRS-R regarding potential psychometric changes and factor structure over time. Analogous analyses are presented for the BDI-II. Although the factor structure of the BDI-II is well studied cross-sectionally, studies testing longitudinal measurement invariance are rare [29]. A focus on the CDRS-R seems justified given the limited knowledge regarding its factor structure [23] and the increasing reliability and homogeneity in factor structure over time, hitherto only reported by Isa et al. [18].

## Methods

### Participants and procedures

The present study consists of a secondary analysis of data from a multicenter, randomized controlled trial (RCT) on the effectiveness of bright light therapy in treating depression in adolescent inpatients (DeLight; [27, 28]). From 2018 to 2020, 248 adolescents aged 12–18 years with a moderate to severe episode of major depressive disorder (MDD) as their main diagnosis and a BDI-II total score > 19 (moderate to severe depression) were screened, and 227 patients were included across four centers. A CDRS-R score ≥ 45 (indicating moderate to severe depression) was used as an additional eligibility criterion. Participants were assessed at four time points: baseline, 4 weeks (post-assessment), 16 weeks (follow-up 1), and 28 weeks (follow-up 2) using a comprehensive test battery and the CDRS-R interview. *N* = 116 participants were randomized to receive bright light therapy and *n* = 111 to placebo red light; 224 patients were analyzed as the intention-to-treat sample and 152 patients (61%) completed the trial. The main reason for loss to follow-up was failure to reach patients after discharge. Participants’ mean age was 15.6 years (SD = 1.5) and 86% were female. For further details on patient characteristics, see Legenbauer et al. [28]. As the RCT showed no significant treatment effects [28], the treatment groups were combined for the following analyses.

### Measures

The revised version of the Beck Depression Inventory (BDI-II [30]) is a 21-item self-assessment tool to assess depression severity in adolescents aged ≥ 13 years and adults. All items are rated on a scale from 0 to 3, with response categories depending on the item. The sum score ranges from 0 to 63, with severity levels characterized as follows: not/minimally depressed (0–13), mildly depressed (14–19), moderately depressed (20–28), and severely depressed (29–63). Reliability and validity are considered to be very good [14, 31].

The Children’s Depression Rating Scale – Revised (CDRS-R [32]) is a semi-structured clinical interview based on DSM-IV criteria that additionally captures three non-verbal symptoms including facial expression. A validated German version is available [24]. With a total of 17 items, the CDRS-R has shown good psychometric properties, including high internal consistency (α = 0.85) and very good interrater reliability [25].

### Statistical analyses

Descriptive and psychometric analyses were conducted using SAS v9.4. To quantify the concordance/discordance between self- and clinician-based ratings, the BDI-II and CDRS-R scores at baseline were z-transformed and the standardized CDRS-R score was subtracted from the standardized BDI-II score^1^. A difference of z < -1 between the two z-scores was classified as “under-reporters” and a difference of z > 1 as “over-reporters” (the cut-off of 1 was used following Dorz et al. [6], and subsequent studies). Thus, under-reporters rated their depression as less severe than their clinician, and over-reporters rated their depression as more severe. All other patients (z >-1 and z < 1) were labelled as “equal reporters” (i.e. concordant).

The psychometric properties of the BDI-II and the CDRS-R were determined by Cronbach’s alpha and item-total correlation (part-whole corrected). For factor analyses, we used principal component analysis (PCA) with promax rotation, as this was the preferred method of analysis for the CDRS-R. Furthermore, we applied the exploratory factor analysis (EFA) for ordered-categorical items provided in M*plus* v7.4 [33] with the mean- and variance-adjusted weighted least squares (WLSMV) estimator and geomin rotation of the factors; confirmatory factor analyses also were performed with M*plus* and WLSMV estimation. The fit of the models was evaluated using the comparative fit index (CFI), the Tucker–Lewis index (TLI), and the root mean square error of approximation (RMSEA). A CFI ≥ 0.95, an RMSEA value ≤ 0.05, and a TLI ≥ 0.95 are considered as indicating a good fit, according to the guidelines of Hu and Bentler [34]. Acceptable fit is indicated for values of CFI ≥ 0.90, and RMSEA ≤ 0.08. Longitudinal measurement invariance was tested by a stepwise comparison of nested confirmatory factor models [11] for the time points baseline (T1) and after four weeks of treatment (T2). In line with our research question, we focused on testing for configural invariance, i.e. equal number of factors and loading patterns over time, and for scalar invariance, i.e., equal factor loadings over time. The configural invariance model (M1) imposes no equality constraints on the parameters, and the number of factors is equal across time. Since factor loadings and item thresholds are interrelated for ordered-categorical indicators (cf. the summary of the discussion in Neufeld et al. [35]), we estimated the combined model used by Neufeld et al. [35] where factor loadings and item thresholds are constrained to be equal across time points and estimated simultaneously (model M2). Furthermore, all models were estimated with correlated residuals for the same item over time, since such autocorrelations should be expected (and have been found in almost all item pairs). Estimated Chi-square statistics of each model are compared with the previous model using the DIFFTEST option in M*plus* [33] to test for equality in model fit.

**Table 1:** Descriptive statistics for BDI-II, CDRS-R, and correlation coefficients between BDI-II and CDRS-R at the four assessment points

Instrument and statistics	T1: baseline	T2: after four weeks	T3: follow-up (16 weeks)	T4: follow-up (28 weeks)
BDI-II (n =	224	199	154	114)
Mean	37.34	29.71	25.49	24.99
Standard deviation	8.68	12.66	14.55	14.33
Cronbach’s alpha	0.814	0.917	0.938	0.938
CDRS-R (n =	224	199	160	121)
Mean	65.25	52.83	48.07	46.99
Standard deviation	10.31	14.94	16.05	16.34
Cronbach’s alpha	0.736	0.891	0.899	0.907
Correlation (n =	224	197	150	107)
r (Pearson)	0.398	0.718	0.734	0.782

## Results

### Correlation between self- and clinician ratings over time

Descriptive statistics of the two measurement instruments are displayed in Table 1 (detailed intervention effects are reported in Legenbauer et al. [28]). For both instruments, mean scores decreased over the time points and standard deviations increased, with the highest change occurring from baseline to post-assessment (four weeks). Internal consistency also increased over time, and again, the strongest increase occurred from baseline to post-assessment. The correlation between BDI-II and CDRS-R scores was 0.40 at baseline, and increased markedly to 0.72 at post-assessment; values were slightly higher at the two follow-ups (16 and 28 weeks). These correlations essentially remained unchanged if Spearman rank correlation coefficients were used (to avoid bias due to non-normal distributions), if only adolescents with BDI-II scores ≥ 20 were included (to select for the initial inclusion criterion with restricted variance), when only analyzing the subgroup of completers, or when analyzing male and female adolescents separately (see supplement 1, table S2 ).

**Table 2:** Correlation coefficients (Pearson/polychoric) between comparable items of the CDRS-R and BDI-II at baseline (T1) and after four weeks of treatment (T2)

CDRS-R Item	BDI-II Item	T1	T2	Difference
1. Impaired schoolwork /concentration and attention	19. Concentration difficulties	0.30 / 0.33	0.50 / 0.58	0.20 / 0.25
2. Difficulty having fun	4. Loss of pleasure	0.19 / 0.20	0.45 / 0.47	0.26 / 0.27
4. Sleep disturbance	16. Changes in sleeping	0.19 / 0.23	0.26 / 0.30	0.07 / 0.07
5. Appetite disturbance	18. Changes in appetite	0.29 / 0.34	0.35 / 0.40	0.06 / 0.06
6. Excessive fatigue	20. Tiredness	0.34 / 0.40	0.49 / 0.53	0.15 / 0.13
8. Irritability	17. Irritability	0.31 / 0.34	0.50 / 0.56	0.19 / 0.22
9. Excessive guilt	5. Guilty feelings	0.37 / 0.43	0.51 / 0.56	0.14 / 0.13
10. Low self-esteem	7. Self-dislike 14. Worthlessness	0.29 / 0.36 0.38 / 0.44	0.52 / 0.59 0.57 / 0.60	0.23 / 0.23 0.19 / 0.16
11. Depressed feelings	1. Sadness	0.32 / 0.37	0.62 / 0.69	0.30 / 0.32
13. Suicidal ideation	9. Suicidal thoughts	0.44 / 0.52	0.58 / 0.69	0.14 / 0.17
14. Excessive weeping	10. Crying	0.19 / 0.20	0.33 / 0.34	0.14 / 0.14

All correlation coefficients at T1 and T2 are significant at *p* < .01. Difference is based simply, and for exploratory purposes, on the difference between values; a difference between z-transformed correlation coefficients might be more informative for statistical testing

Table 2 presents correlations between self- and clinician ratings for items that address approximately the same symptoms. The Pearson correlation coefficients were augmented by polychoric correlation coefficients, where an underlying continuous distribution for each item is assumed. However, differences were small, with the polychoric correlations consistently being slightly higher. All correlation coefficients were significant but differed clearly in size. Table 2 also displays the respective differences between baseline and post-assessment. The increase in correlations was strongest for the items depressive feelings, low self-esteem, and difficulty having fun, while the correlation for somatic symptoms showed little increase. The full matrices of intercorrelations between all items are provided in supplement 2.

### Description of the concordance subgroups

Frequencies for the subgroups according to the difference in z-scores (“under-”, “equal-”, and “over-reporters”) were approximately normally distributed (as can be expected), with 63% for equal reporters and 19% and 18%, respectively, for under- and over-reporters. Subgroup was not significantly associated with gender (Chi² (2) = 1.00, *p* = .607) or age (*F* (2, 221) = 0.53, *p* = .591).

### Predictive value of concordance/discordance for improvement

The difference between under-, equal-, and over-reporters at post-assessment (4 weeks) was not significant for the CDRS-R score (*F* (2, 196) = 2.26, *p* = .107, R² = 0.023) but was significant for the BDI-II score (*F* (2, 196) = 3.57, *p* = .030, R² = 0.036). At the first follow-up (16 weeks), the difference between the three groups on the CDRS-R remained non-significant (*F* (2, 157) = 2.10, *p* = .126, R² = 0.026) and the difference on the BDI-II remained significant (*F* (2, 151) = 5.82, *p* = .004, R² = 0.072). Post-hoc comparison of means (Scheffé test) revealed that under-reporters and equal reporters did not differ from one another at first follow-up, but over-reporters had higher BDI-II scores compared to the other two groups. At post-assessment, only under- vs. over-reporters differed significantly regarding BDI-II scores.

### Psychometric analyses of the CDRS-R

Concerning item-total correlations of the CDRS-R at baseline, the internal consistency was only moderate (Cronbach’s alpha = 0.74; after omitting items 8 and 12, which did not significantly correlate with the sum score, the alpha increased only slightly, to 0.77). At post-assessment (4 weeks), the homogeneity between symptoms was much higher (alpha = 0.89) and almost all items exhibited item-total correlations > 0.40, including items 8 and 12. At the first follow-up (after 16 weeks), a slight increase in item-total correlations was found for many symptoms, but internal consistency increased only minimally (alpha = 0.90). For a full description of item-level means, standard deviations, and item-total correlations, see table S2 in the supplement 1.

### Factor analyses of the CDRS-R

For the baseline data, factor analyses using PCA revealed that three factors were significant according to the parallel analysis (PA) criterion. One factor was constituted by the non-verbal items, but the other two factors were not clearly distinguishable. Regarding EFA, inspection of the goodness-of-fit indices for the solutions with different numbers of factors (table S3) revealed that the assumption of a single underlying factor was not tenable due to insufficient goodness-of-fit values. The solution with two factors showed a close to acceptable fit, but the improvement for the three-factor solution was substantial. Again, one factor consisted primarily of the non-verbal items, while the other two could not be clearly labelled.

At post-assessment (4 weeks), the PCA (PA criterion) and EFA (goodness-of-fit indices, table S3a) favored a two-factor solution. Thus, the two-factor solution was preferred, indicating a distinction between the reported and the observed items. Factor loadings of this solution and the three-factor solution for baseline data are displayed in the supplement 1 (tables S4a and S4b), along with some further results on explained variance and factor intercorrelations, which both increased from baseline to post-assessment.

### Psychometric and factor analyses of the BDI-II

The internal consistency at baseline was relatively low (Cronbach’s alpha = 0.81, Table 1). Item-total correlations for three items were below the commonly used cut-off of 0.30, and only two items were (slightly) above 0.50. At post-assessment, homogeneity between symptoms was much higher (alpha = 0.92); almost all items exceeded the value of 0.30, and 16 items were above 0.50. At the first follow-up (16 weeks), item-total correlations slightly increased for many symptoms and internal consistency increased further to alpha = 0.94, remaining at this level at the second follow-up (28 weeks; Table 1).

The factor analyses (PCA) for the BDI-II revealed that two factors were significant according to the PA criterion at baseline; the first factor explained 22.1% of the variance. At post-assessment, homogeneity increased remarkably, as only one factor was significant according to the PA criterion and the first factor explained 39.4% of the variance. Confirmatory factor analyses showed that goodness-of-fit indices increased from baseline to post-assessment and a one-factor solution would be acceptable at post-assessment although the two-factor structure comprising a cognitive and an affective/somatic factor was still preferable. Moreover, the factor intercorrelation rose from 0.65 at baseline to 0.84 at post-assessment, also indicating increased homogeneity (see complete results in supplement 1, table S3c, and subscale-specific results in table S5).

The testing for configural invariance (M1) revealed acceptable to good goodness-of-fit criteria for the one-factor solution and good fit for the two-factor solution (Table 3), i.e. configural invariance over time could be assumed for both factor solutions. The restrictions for equal loadings and thresholds (M2) decreased goodness-of-fit criteria only marginally, but the differences in the DIFFTEST indicated that the scalar invariance model (M2) fit the data significantly worse than the configural invariance model (M1). Thus, the assumption of equal loadings and thresholds over time had to be rejected for both factor solutions.

**Table 3:** Model comparison for the BDI-II for longitudinal invariance from baseline (T1) to after four weeks (T2) for the one-factor and the two-factor model

Model	Chi^2^	df	CFI	TLI	RMSEA (90%-CI)	Diff.-Test
One factor						
Model						
M1: configural invariance	1200.21	797	0.927	0.922	0.048 (0.042 − 0.053)	-
M2: scalar (equal loadings and thresholds)	1294.92	858	0.921	0.921	0.048 (0.042 − 0.053)	chi²=123.99, df = 61, *p* = .000
Two factors (Cog and Som)						
Model						
M1: configural invariance	1044.54	792	0.955	0.951	0.038 (0.031 − 0.044)	-
M2: scalar (equal loadings and thresholds)	1137.92	851	0.948	0.948	0.039 (0.033 − 0.045)	chi²=113.75, df = 59, *p* = .000

Cog: cognitive factor including items 2, 3, 5–9, and 14; Som: somatic-affective factor including items 1, 4, 10–13, and 15–21, following the assignment of an often-used two-factor solution (c.f [19])

## Discussion

The present study explored the concordance of self- and clinician-rated depression severity over the course of treatment in a large sample of adolescents during inpatient therapy. The findings for the main research questions can be summarized as follows:

1. The correlation between self- and clinician-rated depression (BDI-II and CDRS-R) was low to moderate, with *r* = .40 at baseline.
2. The correlation between self- and clinician-rated depression clearly increased across the assessment points, with the strongest increase from baseline to post-assessment (four weeks after baseline).
3. The predictive value of initial concordance/discordance was low when comparing subgroups with high initial differences (under- or over-reporters) with adolescents with little initial difference (equal reporters).

### Predictive value of concordance/discordance for improvement

The initial differences between under-, equal-, and over-reporters seemed to disappear after four weeks, as there were no significant differences in the CDRS-R at post-assessment. Differences in the BDI-II were significant but with a small effect size. Furthermore, the subgroups did not differ regarding gender, which is in line with other studies [5, 6, 9]. While Dunlop et al. [8] found that female patients were more likely to overrate their depression severity relative to the clinician, this discrepancy disappeared after the acute-phase treatment.

### Concordance between self- and clinician ratings over time

The moderate correlation between the BDI-II and CDRS-R at baseline and the clear increase after four weeks correspond well with the findings reported by Bukumiric et al. [1] for studies in adults. An often-mentioned reason for this increase is the increase in standard deviations, i.e. the distribution of the sum scores becomes larger. Although this argument appears reasonable, its explanatory power seems limited. For example, the correlation for the subgroup of adolescents who still showed moderate depression (BDI-II ≥ 20) likewise increased, and other methodological aspects, such as increasing homogeneity and change in factor structure, provide greater insight (see discussion below). As with our earlier study [5] as well as research in adults [9], we did not observe gender-related differences in correlations (see supplement 1, table S1), but the general association is unclear, as the meta-analysis by Bukumiric et al. [1] did not include gender.

Regarding the increased correlations on the item level, one might argue that the item-level correlations may also be subject to increasing variance, but the differences in the increase suggest a more differentiated picture. Specifically, the increase was much higher for mood and cognitive items (sadness, self-esteem, difficulty having fun) than for items in the more somatic realm (sleep and appetite problems). Suicidality showed only a moderate increase, but its concordance was already highest at baseline and remained high after four weeks. This confirms the findings of Straub et al. [5], who therefore considered self-report of suicidality as useful in clinical practice. The finding that concordance regarding affective-cognitive symptoms (and especially for the core symptoms of depression) improves more than concordance regarding somatic symptoms is contrary to expectation and needs to be confirmed in future research, as there are no comparable results so far in the literature.

Furthermore, the correlations between items addressing the same symptom realm were higher than those between other items, in line with previous findings [5]. Interestingly, we did not find a generally low-to-moderate correlation between all items, as might be expected given the high internal consistency (after four weeks) of each instrument alone.

### Psychometric analyses of the CDRS-R

Internal consistency of the CDRS-R at baseline was only sufficient (according to the common classification of Cronbach’s alpha), but corresponds to the study by Isa et al. [18], who reported alpha = 0.64 at the first assessment. Concerning specific items, item-total correlations of several items were low or near zero, especially item 8 (irritability) and item 12 (morbid ideations). Isa et al. [18] did not provide item-total correlations, but in a partly overlapping sample, the value for irritability was 0.11 at baseline [17]. These findings are surprising and in contrast to the clinical sample in the US manual [32] as well as the Keller et al. [25] study, in which most item-total correlations were clearly higher (including for irritability and morbid ideation, with 0.52 and 0.54, respectively) and the alpha lay at 0.90.

After four weeks, the internal consistency increased considerably, reaching a good to very good level, again in line with Isa et al. [18], and item-total correlations were acceptable to good for all items, as was the case in the exit session in the study by Mayes et al. [17]. The values remained at this level at the later assessment points. Currently, the low item-total correlations of some items at baseline cannot be reasonably explained, but one may speculate that in a sample with at least moderate depression severity, some items are no longer very discriminatory. Thus, from the clinician’s perspective, the degree of irritability is not related to the severity of depression.

### Psychometric analyses of the BDI-II

The psychometric properties of the BDI-II after four weeks and at later time points resembled the results reported in other studies, although the internal consistency at baseline lay in the lower range of Cronbach’s alphas reported in a comprehensive review [20]. Thus, homogeneity in the BDI-II (reflected by reliability and a shift towards a one-factor solution) also seemed to be dependent on assessment time, as also found in previous studies with adolescents [14, 15], but to a much lesser extent than the CDRS-R. A similar decrease in factor structure was found for the construct working alliance in psychotherapy for generalized anxiety disorder [36]. Over a series of psychotherapeutic sessions, the better session-level outcome clients switched from three factors to an integrated single factor conceptualization of the therapeutic alliance, thus indicating that the majority of patients evolve their concept of the alliance overtime [36].

### Factor analyses of the CDRS-R

The factor structure at baseline comprised three factors that were difficult to clearly distinguish by content. This difficult-to-interpret structure corresponds to previous studies, which suggested even more factors at baseline [18, 21]. In both studies, the majority of participants were children, and the results may be influenced by age, although Keller et al. [25] found no relevant differences in factor loadings between children and adolescents in a school sample. After four weeks, a two-factor solution emerged, comprising a factor reflecting the reported symptoms and a factor reflecting the observed symptoms, again in accordance with Isa et al. [18]. The factor intercorrelation was likewise very similar, at 0.48 in our sample and 0.52 in Isa et al. [18]. Although the determination of the factor structure of the CDRS-R still requires further research [23], it has been fairly consistently found that the factor observed is frequently linked to items 2 and 3 (difficulty having fun and social withdrawal) in clinical samples ( [18], supplement 1, table S4a) and in school samples [25]. This item pair, termed as factor anhedonia by Guo et al. [21], thus seems more strongly related to the observed items, at least if a two-factor solution is chosen in the PCA.

Isa et al. [18] discussed several potential reasons for the increasing homogeneity in CDRS-R ratings. In particular, patients may exhibit a halo effect, i.e., the tendency to answer more globally and according to their current mood. Moreover, they may gain greater insight into their depression, viewing it as a unified whole rather than a set of minimally related symptoms. Furthermore, depression treatment may reduce some symptoms more than others, if different symptoms respond to treatment to differing degrees. While all of these alternatives can lead to a simplification of the factor structure via different mechanisms, they cannot be evaluated further as information on the specific psychotherapy components is lacking [18].

### Examining the assumption of longitudinal invariance of self- and clinician ratings

The longitudinal measurement invariance of the CDRS-R was not evaluated further, as even the first prerequisite, the assumption of the same factor structure over time (configural invariance), seemed to be violated. Our results show, in line with Isa et al. [18], that the number of factors and the factor structure clearly changes over the first assessment points. After four weeks, the assumption of a two-factor structure seems plausible, and tests of longitudinal invariance regarding equal factor loadings (metric invariance) and further restricted models could be evaluated to compare later time points; however, this seems less relevant for treatment studies in which the main emphasis is on intervention effects from baseline to the next assessment points after therapy onset.

For the BDI-II, configural invariance could be assumed (regardless of whether a one- or two-factor solution is chosen), but the assumption of equality of factor loadings had to be rejected for both solutions. Similarly, Elhai et al. [12] found that factor loadings in a three-factor solution differed significantly between two time points, with larger factor loadings on average after one month of treatment. Wu [15] found no significant difference in factor loadings between two measurement points in a sample of depressed adolescents before and after 16–18 weeks of individual counseling and cognitive behavioral therapy. However, the thresholds differed between time points, indicating a recalibration of symptoms [15]^2^. Using an item response theory (IRT) approach, Keller & Alexandrowicz [37] also revealed in a large sample of adult psychosomatic inpatients that the discrimination parameters (which can be seen as equivalent to factor loadings) in all BDI-II items increased from admission to discharge assessment.

Furthermore, stronger factor intercorrelations at the second time point, as found in our data and in other studies [10, 15], may be interpreted as reflecting a reconceptualization of the construct, i.e. patients seem to view depression as a more unified concept after treatment [10]. The testing of specific confirmatory factor models allows the type of response shift to be determined (see, e.g., Sebille et al. [38], for an overview on terminology and discussion of methods) and can be used to correct the latent means at the second administration. Applications of the corrections in latent means revealed mixed results with an overestimation [10] or an underestimation [15] of depressive symptomatology after treatment compared to before treatment, or a slight underestimation in improvement in one treatment group over time [39].

While a reconceptualization seems plausible for self-reports, this argument can hardly be appropriate for clinician reports. Raters are trained in diagnostics and receive training on the CDRS-R interview before the start of a study; while they may become more experienced over the course of a study, studies often last for several years and patients are assessed on a continuous basis. Furthermore, the number of raters is usually small, and raters are independent from patients, at least in controlled treatment studies. Most articles provide information on the experience and profession of raters, but the number of raters is rarely reported or clear^3^ (cf. Stallwood et al. [23], Table 1), and to the best of our knowledge, no analysis has considered the nested structure (ratings nested within rater and raters nested within centers).

Interestingly, the reasons for increasing homogeneity discussed by Isa et al. [18] are seen in the patient, while the rater is deemed as objective and the role of his/her own interpretation is neglected. Only after many diagnostic sessions or in naturalistic studies where the rating clinician also operates as the treating psychotherapist might discrepancies be reduced for the following reasons [8]: (1) greater shared understanding with the clinician regarding symptom descriptions; (2) enhanced ability to concentrate due to improvement over time; (3) reduced patient mood bias in recall of the symptoms; or (4) increased disclosure to the clinician (better “rapport”). The masking of clinicians was found to be an important predictor of the differences between patient- and clinician-rated outcomes [40].

## Limitations

To calculate the correlation coefficients between the BDI-II and CDRS-R, we used the available sample size at each time point. Imputation of missing values might change results, but for the (most important) first two time points, the dropout rate was only about 10%. The correlation coefficients for all four time points were also calculated for the subsamples of completers and subsequent dropouts, and essentially remained unchanged.

Factor analyses of the CDRS-R revealed increasing homogeneity in general, but the factor loadings for baseline data were ambiguous and there were major differences in some loadings between EFA and PCA solutions, which remained after four weeks. These results require replication.

## Conclusion

Concordance between self- and clinician report increased over the course of treatment, as previously reported in some studies with adults. This effect was accompanied by an increase in homogeneity and factor loadings as well as increasing factor intercorrelations in both instruments, indicating that some type of response shift (reprioritization and reconceptualization) seems to have occurred. Since the current study is the first to analyze the correlation between self- and clinician report over time in depressed adolescents and the time-related psychometric properties of the two instruments, future research is needed to replicate these findings in other samples and, especially for the CDRS-R, to gain more insight into the factor structure and factor loadings, preferably taking into account the nested structure of ratings within raters and potentially clinic-specific differences. Concerning clinical consequences and study planning, our study revealed (supplement 1, table S1) that the clinician report (CDRS-R) yielded larger effect sizes for improvement than the self-report, in line with a meta-analysis in adults, in which clinician-rated outcomes were significantly more sensitive to change [39]. However, it can also be argued that self-report may be more conservative and it is therefore probably optimal to include both informants in clinical trials [2, 41].

## Supplementary Information

Below is the link to the electronic supplementary material.

Supplementary Material 1.

Supplementary Material 2.

## Data Availability

The data that supports the findings of this study are available from the corresponding author upon reasonable request.

## Footnotes

1 The difference z(BDI-II) – z(CDRS-R), or more generally “self – clinician”, was used in most previous studies and in our earlier publication [5]. However, the sign of the difference is arbitrary.

2 There is debate regarding whether loadings and thresholds can be tested separately in the case of ordinal-categorical variables (c.f [35]) , thus calling the interpretation of this finding into question.

3 In the current study, almost all CDRS-R were assessed by 1–2 external raters per center.
