# Supplementary material for: Concordance between self- and clinician ratings of depression during inpatient treatment in adolescents: changes over time and probable response shift
Source: Child Adolesc Psychiatry Ment Health. 2025 Nov 15;19:125. doi: 10.1186/s13034-025-00993-3 (PMC12619375; doi:10.1186/s13034-025-00993-3)
Supplement: Supplementary file 1 — Supplementary Material 1. [file 13034_2025_993_MOESM1_ESM.docx]

**Supplementary material 1 for:***Keller/Holtmann/Kölch/Legenbauer: Concordance between self- and clinician ratings of depression during inpatient treatment in adolescents: Changes over time and probable response shift*

Table S1: Descriptive statistics for BDI-II, CDRS-R, and correlation coefficients between BDI-II and CDRS-R at the four assessment points (long version of table 1).

| Instrument and statistics | T1: baseline | T2: after four weeks | T3: follow-up (16 weeks) | T4: follow-up (28 weeks) |  |
| --- | --- | --- | --- | --- | --- |
| BDI-II (n = ) | 224 | 199 | 154 | 114 |  |
| Mean | 37.34 | 29.71 | 25.49 | 24.99 |  |
| Standard deviation | 8.68 | 12.66 | 14.55 | 14.33 |  |
| Median | 37.00 | 30.00 | 25.50 | 26.00 |  |
| Cronbach’s alpha | .814 | .917 | .938 | .938 |  |
| Effect size | - | -0.71 | -1.02 | -1.07 |  |
| CDRS-R (n = ) | 224 | 199 | 160 | 121 |  |
| Mean | 65.25 | 52.83 | 48.07 | 46.99 |  |
| Standard deviation | 10.31 | 14.94 | 16.05 | 16.34 |  |
| Median | 64.50 | 52.00 | 46.25 | 45.30 |  |
| Cronbach’s alpha | .736 | .891 | .899 | .907 |  |
| Effect size | - | -0.98 | -1.30 | -1.37 |  |
|  |  |  |  |  |  |
| Correlation (n = ) | 224 | 197 | 150 | 107 |  |
| r (Pearson) | .398 | .718 | .734 | .782 |  |
| r (Spearman) | .393 | .694 | .735 | .778 |  |
|  |  |  |  |  |  |
| *Correlation in subgroups:* |  |  |  |  |  |
| BDI-II ≥ 20 at Tx (n = ) | 224 | 154 | 89 | 67 |  |
| r (Pearson) | .398 | .650 | .503 | .650 |  |
| r (Spearman) | .393 | .610 | .477 | .641 |  |
| Completers (n = ) | 152 | 151 | 119 | 81 |  |
| r (Pearson) | .418 | .710 | .704 | .797 |  |
| r (Spearman) | .417 | .679 | .704 | .791 |  |
| Female adolescents (n = ) | 192 | 165 | 127 | 94 |  |
| r (Pearson) | .384 | .727 | .729 | .790 |  |
| r (Spearman) | .389 | .707 | .722 | .795 |  |
| Male adolescents (n = ) | 32 | 32 | 23 | 13 |  |
| r (Pearson) | .388 | .648 | .765 | .550 |  |
| r (Spearman) | .335 | .579 | .725 | .546 |  |

*Note:* Effect sizes were computed with averaged standard deviations (formula 10 in Lakens, 2013), i.e. without taking correlations between time points into account. Differences in correlations between female and male adolescents were small and non-significant for T1 – T3; for T4, the difference was larger but was non-significant due to the small sample size of males (*z* = 1.36, *p* = .087).

Table S2: Means (M), standard deviations (SD) and item-total correlation coefficients (r_it_) for the CDRS-R at three time points (*n* = 224, 199, 160).

|  | T1: baseline | |  | T2: after four weeks | |  | T3: follow-up (16 weeks) | |
| --- | --- | --- | --- | --- | --- | --- | --- | --- |
| CDRS-R Item | M (SD) | r_it_ |  | M (SD) | r_it_ |  | M (SD) | r_it_ |
| 1. Impaired schoolwork | 3.90 (1.58) | .36 |  | 2.92 (1.43) | .40 |  | 2.94 (1.66) | .47 |
| 2. Difficulty having fun | 4.00 (1.42) | .47 |  | 2.91 (1.47) | .65 |  | 2.68 (1.54) | .70 |
| 3. Social withdrawal | 3.88 (1.39) | .35 |  | 2.91 (1.37) | .61 |  | 2.62 (1.61) | .54 |
| 4. Sleep disturbance | 4.31 (1.01) | .16 |  | 3.23 (1.38) | .51 |  | 2.98 (1.50) | .61 |
| 5. Appetite disturbance | 3.20 (1.23) | .33 |  | 2.59 (1.15) | .42 |  | 2.38 (1.28) | .46 |
| 6. Excessive fatigue | 4.66 (1.57) | .32 |  | 3.70 (1.71) | .54 |  | 3.63 (1.79) | .54 |
| 7. Physical complaints | 3.18 (1.48) | .30 |  | 2.49 (1.39) | .38 |  | 2.20 (1.40) | .30 |
| 8. Irritability | 3.42 (1.50) | .02 |  | 2.94 (1.42) | .42 |  | 2.66 (1.41) | .52 |
| 9. Excessive guilt | 3.96 (1.45) | .25 |  | 3.35 (1.58) | .52 |  | 2.89 (1.46) | .53 |
| 10. Low self-esteem | 4.69 (1.44) | .30 |  | 4.03 (1.58) | .60 |  | 3.57 (1.62) | .68 |
| 11. Depressed feelings | 4.78 (1.13) | .63 |  | 3.97 (1.48) | .79 |  | 3.23 (1.55) | .80 |
| 12. Morbid ideation | 3.93 (1.60) | .07 |  | 3.38 (1.69) | .42 |  | 2.89 (1.44) | .48 |
| 13. Suicidal ideation | 4.61 (1.49) | .44 |  | 3.40 (1.70) | .67 |  | 3.02 (1.73) | .55 |
| 14. Excessive weeping | 4.62 (1.72) | .45 |  | 3.73 (1.86) | .69 |  | 3.03 (1.79) | .68 |
| 15. Depressed facial affect | 3.37 (1.19) | .45 |  | 2.90 (1.27) | .67 |  | 2.38 (1.28) | .63 |
| 16. Listless speech | 2.37 (0.92) | .33 |  | 2.18 (0.90) | .44 |  | 2.06 (0.94) | .50 |
| 17. Hypoactivity | 2.37 (1.16) | .37 |  | 2.09 (1.01) | .47 |  | 1.85 (0.87) | .48 |
| Sum score / Cronbach’s alpha | 65.3 (10.3) | .74 |  | 52.8 (14.9) | .89 |  | 48.1 (16.1) | .90 |

*Note:* CDRS-R items are rated from 1 – 7, except for items 4, 5, and 16, which are rated from 1 – 5.

Table S3a: Fit indices for exploratory factor analyses of the CDRS-R with 1 – 3 factors at baseline and after four weeks.

| Model | *Chi*^2^ | *df* | CFI | TLI | RMSEA (90%-CI) |
| --- | --- | --- | --- | --- | --- |
| T1: baseline |  |  |  |  |  |
| 1 factor | 400.8 | 119 | .782 | .751 | .103 (.092 - .114) |
| 2 factors | 221.2 | 103 | .909 | .879 | .072 (.059 - .085) |
| 3 factors | 133.0 | 88 | .965 | .946 | .048 (.030 - .064) |
| T2: after four weeks |  |  |  |  |  |
| 1 factor | 392.3 | 119 | .894 | .879 | .107 (.096 - .119) |
| 2 factors | 215.4 | 103 | .956 | .942 | .074 (.060 - .088) |
| 3 factors | 129.8 | 88 | .984 | .975 | .049 (.030 - .066) |

Table S3b: Fit indices for confirmatory factor analyses of the CDRS-R at baseline and after four weeks.

| Model | *Chi*^2^ | *df* | CFI | TLI | RMSEA (90%-CI) |
| --- | --- | --- | --- | --- | --- |
| T1: baseline |  |  |  |  |  |
| 1 factor | 400.8 | 119 | .782 | .751 | .103 (.092 - .114) |
| 2 factors | 279.6 | 118 | .875 | .856 | .078 (.066 - .090) |
| Bifactor (G + 2S) | 167.7 | 102 | .949 | .932 | .054 (.039 - .068) |
| T2: after four weeks |  |  |  |  |  |
| 1 factor | 392.3 | 119 | .894 | .879 | .107 (.096 - .119) |
| 2 factors | 263.5 | 118 | .944 | .935 | .079 (.066 - .081) |
| Bifactor (G + 2S) | 181.4 | 102 | .969 | .959 | .063 (.047 - .077) |

*Note:* The two factors and 2S in the bifactor model were “reported” (items 1 – 14) and “observed” (items 15 – 17).

Table S3c: Fit indices for confirmatory factor analyses of the BDI-II at baseline and after four weeks.

| Model | *Chi*^2^ | *df* | CFI | TLI | RMSEA (90%-CI) |  |
| --- | --- | --- | --- | --- | --- | --- |
| T1: Baseline |  |  |  |  |  |  |
| one factor | 429.5 | 189 | .842 | .825 | .075 (.066 - .085) | - |
| two factors (Cog + Som) | 321.5 | 188 | .912 | .902 | .056 (.046 - .067) | r_12_ = .645 |
| T2; After four weeks |  |  |  |  |  |  |
| one factor | 443.0 | 189 | .938 | .931 | .082 (.072 - .092) | - |
| two factors (Cog + Som) | 355.4 | 188 | .959 | .954 | .067 (.056 - .077) | r_12_ = .840 |

*Note:* Cog: cognitive factor including items 2, 3, 5–9, and 14; Som: somatic-affective factor including items 1, 4, 10–13, and 15–21, following the assignment of an often-used two-factor solution (c.f. Keller et al., 2020).

Table S4a: Factor loadings (PCA) for the CDRS-R items at baseline and after four weeks.

|  | T1: baseline  3-factor solution | | |  | T2: after four weeks  2-factor solution | |  |
| --- | --- | --- | --- | --- | --- | --- | --- |
| CDRS-R item | F1 | F2 | F3 |  | F1 | F2 |  |
| 1. Impaired schoolwork |  |  | **.67** |  |  | .38 |  |
| 2. Difficulty having fun | .47 |  | .46 |  | .24 | **.60** |  |
| 3. Social withdrawal | .26 | .21 |  |  | .29 | **.51** |  |
| 4. Sleep disturbance |  | **.52** |  |  | .**56** |  |  |
| 5. Appetite disturbance |  | **.51** |  |  | .49 |  |  |
| 6. Excessive fatigue |  |  | **.67** |  | **.54** |  |  |
| 7. Physical complaints |  | .22 | .42 |  | **.59** |  |  |
| 8. Irritability | -.28 |  | **.57** |  | .43 |  |  |
| 9. Excessive guilt |  | **.51** |  |  | **.80** |  |  |
| 10. Low self-esteem | .31 |  | .32 |  | .34 | .48 |  |
| 11. Depressed feelings | .39 | .43 | .23 |  | **.50** | .46 |  |
| 12. Morbid ideation | -.35 | **.60** |  |  | **.70** |  |  |
| 13. Suicidal ideation |  | **.55** |  |  | .43 | .45 |  |
| 14. Excessive weeping |  | **.72** |  |  | **.54** | .33 |  |
| 15. Depressed facial affect | **.89** |  |  |  |  | **.82** |  |
| 16. Listless speech | **.84** |  | -.24 |  | -.27 | **.90** |  |
| 17. Hypoactivity | **.76** |  |  |  | -.22 | **.86** |  |

*Note:* Only factor loadings ≥ |.20|.

Further results with PCA:
T1: Eigenvalues: 3.86, 1.82, 1.50, 1.20, 1.02, …; %Variance cumulative: 22.7, 33.4, 42.2, 49.3, …; inter-factor correlations: .27, .26, .23.
T2: Eigenvalues: 6.54, 1.59, 1.24, … ; %Variance cumulative: 38.5, 47.9, 55.1, …; inter-factor correlation = .48

Table S4b: Factor loadings (EFA, WLSMV estimator) for the CDRS-R items at baseline and after four weeks.

|  | T1: baseline  3-factor solution | | |  | T2: after four weeks  2-factor solution | |  |
| --- | --- | --- | --- | --- | --- | --- | --- |
| CDRS-R item | F1 | F2 | F3 |  | F1 | F2 |  |
| 1. Impaired schoolwork | **.66** |  |  |  | .39 |  |  |
| 2. Difficulty having fun | **.52** | .32 |  |  | **.51** | .30 |  |
| 3. Social withdrawal | .23 |  | .18 |  | **.54** | .20 |  |
| 4. Sleep disturbance |  |  | .41 |  | **.57** |  |  |
| 5. Appetite disturbance |  |  | .38 |  | .48 |  |  |
| 6. Excessive fatigue | **.50** |  |  |  | **.60** |  |  |
| 7. Physical complaints | .31 |  | .18 |  | **.51** |  |  |
| 8. Irritability | .26 | -.19 |  |  | **.52** |  |  |
| 9. Excessive guilt |  |  | .39 |  | **.81** | -.33 |  |
| 10. Low self-esteem | .28 | .20 |  |  | **.53** | .22 |  |
| 11. Depressed feelings | .26 | .28 | **.51** |  | **.81** |  |  |
| 12. Morbid ideation |  | -.21 | .42 |  | **.59** | -.20 |  |
| 13. Suicidal ideation |  |  | .48 |  | **.68** |  |  |
| 14. Excessive weeping |  |  | **.76** |  | **.77** |  |  |
| 15. Depressed facial affect |  | **.85** |  |  | .33 | **.67** |  |
| 16. Listless speech |  | **.84** |  |  |  | **.84** |  |
| 17. Hypoactivity |  | **.68** |  |  |  | **.65** |  |

*Note:* Only significant factor loadings (p < .05). Inter-factor correlations at T1: .29, .28, .29; at T2: .50.

*Correlations between subscales in a confirmatory factor model*

If a two-factor model is imposed at both time points, the two CDRS-R subscales “reported” (items 1 – 14) and “observed” (15 – 17) were correlated at *r* = .59 at baseline and the correlation increased to .71 after four weeks.

With a structural equation model, i.e., corrected for measurement error, the two CDRS-R subscales “reported” and “observed” correlated with the BDI-II total score at *r* = .504 (reported) and .210 (observed) at baseline. After four weeks, the latent correlation increased to .786 and .571, respectively.

Table S5: Reliability (Cronbach’s alpha) for subscales and variance explained for factors of the BDI-II and the CDRS-R at the four assessment points.

| Instrument and statistics for subscales/factors | T1: baseline | T2: after four weeks | T3: follow-up (16 weeks) | T4: follow-up (28 weeks) |  |
| --- | --- | --- | --- | --- | --- |
| BDI-II |  |  |  |  |  |
| Cronbach’s alpha |  |  |  |  |  |
| Cognitive | .769 | .884 | .911 | .908 |  |
| Somatic/affective | .719 | .858 | .885 | .885 |  |
|  |  |  |  |  |  |
| Variance explained |  |  |  |  |  |
| Factor 1 | 22.1% | 39.4% | 47.3% | 47.4% |  |
| Factor 2 | 9.5% | 7.5% | 6.9% | 6.4% |  |
|  |  |  |  |  |  |
| CDRS-R |  |  |  |  |  |
| Cronbach’s alpha |  |  |  |  |  |
| Reported | .694 | .877 | .888 | .892 |  |
| Observed | .790 | .805 | .820 | .863 |  |
|  |  |  |  |  |  |
| Variance explained |  |  |  |  |  |
| Factor 1 | 22.7% | 38.5% | 41.7% | 45.9% |  |
| Factor 2 | 10.7% | 9.4% | 11.0% | 14.1% |  |
| Factor 3 | 8.9% | 7.3% | 8.8% | 7.2% |  |

*Note:* For subscale composition see tables S3b and S3c; the third factor for the CDRS-R at T2, T3 and T4 is not significant, and variance explained is shown for completeness.

**Supplement text: Sayer et al. – approach for subgroup outcome**

Sayer et al. (1993) suggested building subgroups below or above the median of the two assessment instruments (HAMD and BDI) at baseline. Thus, four combinations emerged, e.g. a subgroup with low HAMD and high BDI. Analysis of the change scores revealed that the high HAMD / low BDI subgroup showed the greatest improvement on the HAMD, with an effect size that was approximately twice that of the other three subgroups combined (Sayer et al., 1993).

Standardized scores (z-scores) were used to build the four combinations of BDI-II and CDRS-R according to being above or below z = 0 (which is equivalent to being above or below the mean value), respectively. For example, the subgroup with the combination z(BDI-II) < 0 and z(CDRS-R) > 0 was denoted as “low BDI-II/high CDRS-R”.

The initially discordant or concordant subgroups based on the four combinations of BDI-II and CDRS-R (based on being above or below the respective mean values at T1 baseline) showed subgroup frequencies in the range of 19% to 33%. Gender was not significantly related to subgroup (Chi² (3) = 4.30, *p* = .231) and the subgroups did not differ in age (*F* (3, 220) = 1.15, *p* = .331).

The mean differences at T2 (post-treatment) of the four combination subgroups revealed significant differences for the BDI-II (*F* (3, 195) = 25.70, *p* < .0001, R² = .283) and for the CDRS-R (*F* (3, 195) = 19.23, *p* < .0001, R² = .228). For prediction of T3 (12-week follow-up), the four subgroups continued to show significant differences (p < .0001) in both assessment instruments.

The significant differences between the four combinations seem to reflect the initial differences in depression severity between groups at baseline, which basically persist.

**Supplement text: correlation on the subscale level**

*Correlation on the subscale level*

Sayer et al. (1993) used the PCA to determine factors (5 for the BDI, 8 for the HAMD) leading to, e.g., somatic and cognitive/affective subscales in the HAMD and the BDI. If the two instruments differed in the number of cognitive and somatic items (which was discussed as a reason for the low correlation), one might expect higher correlations between factors reflecting the same item content. However, the sum scores of the corresponding factors (and of every factor pair) did not show higher correlations than the total scale scores. Thus, the differences in the sampling of depressive symptoms appeared not to contribute to the relatively low baseline correlation (Sayer et al., 1993).

The subscale correlations in the adolescent sample of Straub et al. (2014), which were based on the five factors described by Guo et al. (2006), also did not exceed the total score correlation between the BDI-II and the CDRS-R, i.e. neither the cognitive factors nor the somatic factors, where a superior correlation was expected, showed a stronger association.

**Supplemental Results**

*Cross-informant correlations for baseline and post-assessment (four weeks)*

In a complementary predictive analysis for the total sample, we analyzed the correlations between the two instruments over time as “predictors” from T1 (baseline) to T2 (after four weeks). The correlation between the CDRS-R at T1 and T2 was *r* = .54 and the correlation between the BDI-II at T1 and T2 was *r* = .55 (both p < .0001). The cross-informant correlations were still significant: CDRS-R at T1 and BDI-II at T2: *r* = .36, and BDI-II T1 and CDRS-R T2: *r* = .35 (both p < .0001). Thus, adolescents with higher initial depression scores tended to have higher scores at T2 within the same instrument, but cross-informant correlations were still predictive as well, with a moderate effect size.

*Concordance of change scores in self- and clinician rating (T2 – T1)*

The correlation between the change score (T2 – T1) in the BDI-II and the change score in the CDRS-R, was r = .56. The CGI-Improvement value at T2 showed a high correlation with the change score in the CDRS-R (*r* = .80) and a moderately high correlation with the BDI-II change score (*r* = .49; all *r* significant at p < .0001).

Additional analyses for the change scores revealed that the change scores were related to the respective T1 values, but with a small effect size (BDI-II: *r* = -.15, p = .033; CDRS-R: *r* = -.17, p = .015). Thus, higher initial values tended to be associated with higher change scores (as higher change is more negative = more improvement). For T2, these correlations were high, at *r* = .74 for both instruments.

The correlation between the CGI-I and the change score in the BDI-II (*r* = .49) exceeds the correlation of .36 that was reported by Kaiser et al. (2022). The latter authors used the Global Assessment of Functioning (GAF) as the clinician rating and found a low correlation between changes in the GAF and changes in the BDI-II (*r* = -.24), which is also quite different from our finding of r = .56 and shows that the GAF is not as specific for depression compared to the CDRS-R.

**References** (in addition to the references in the article):

Kaiser T, Herzog P, Voderholzer U, Brakemeier E-L (2022) Out of sight, out of mind? High discrepancy between observer- and patient-reported outcome after routine inpatient treatment for depression. J Affect Disord 300:322–325. [doi.org/10.1016/j.jad.2022.01.019](https://doi.org/10.1016/j.jad.2022.01.019)

Lakens D (2013) Calculating and reporting effect sizes to facilitate cumulative science: a practical primer for t-tests and ANOVAs. Front Psycholog 4:863. doi:10.3389/fpsyg.2013.00863
